# Methods for the Development of Healthcare Practice Recommendations Using Systematic Reviews and Meta-Analyses

**DOI:** 10.3389/fneur.2021.699968

**Published:** 2021-07-08

**Authors:** Thomas Platz

**Affiliations:** ^1^BDH-Klinik Greifswald, Institute for Neurorehabilitation and Evidence-Based Practice, “An-Institut,” University of Greifswald, Greifswald, Germany; ^2^Neurorehabilitation Research Group, University Medical Centre, Greifswald, Germany; ^3^Special Interest Group Clinical Pathways, World Federation for NeuroRehabilitation, North Shields, United Kingdom

**Keywords:** evidence, review – systematic, guideline [MeSH], recommendation, clinical decision

## Abstract

Quality of healthcare can be improved when the best external evidence available is integrated in clinical decision-making in a systematic explicit manner. With the rapid expansion of clinical evidence, the opportunities for evidence-based high-quality healthcare increase. Paradoxically, the likelihood of any one person to get a complete and balanced picture of the evidence available decreases. This is especially true for rehabilitation interventions that are complex in nature and where clinical research is rather diverse. Given the complex nature of the evidence, there is a substantial risk of misinterpreting the complex information both at the level of individual sources (e.g., reports of clinical trials) and for aggregated data syntheses (e.g., systematic reviews and meta-analyses). These risks are inherent in these sources themselves and are in addition related to the methodological expertise necessary to make valid use of the evidence for clinical decision-making. Taken together, there is a great demand for systematic structured guidance from evidence to clinical decision. This methodology paper describes a structured process for the development and report of evidence-based clinical practice recommendations that uses systematic reviews and meta-analyses as evidence source. It provides a comprehensive framework with specific requirements for the development group, the formulation of the healthcare question addressed, the systematic search for the evidence, its critical appraisal, the extraction and the outcome-centered presentation of the evidence, the rating of its quality, strengths and weaknesses, any further considerations relevant for decision-making, and an explicit recommendation statement along with its justification, implementation, and resource aspects. The suggested methodology uses international standards in evidence synthesis, critical appraisal of systematic reviews, rating the quality of evidence, characteristics of recommendations, and guideline development as developed by Cochrane, GRADE (Grading of Recommendations Assessment, Development and Evaluation), AMSTAR (A MeaSurement Tool to Assess systematic Reviews), and AGREE (Appraisal of Guidelines for REsearch & Evaluation). An added distinctive feature of the methodology is to focus on the most up-to-date, most valid evidence and hence to support the development of valid practice recommendations in an efficient way. Practice recommendations generated by such a valid methodology would be generally applicable and promote evidence-based clinical practice globally.

## Introduction

Globally, disabilities affecting everyday life, occupational and other social activities, and hence participation restrictions have dramatically been increasing for the last decades (1), a tendency that is foreseen to continue in the near future.

Persons with health conditions that cause limitations in functioning can benefit from rehabilitation. The need for rehabilitation continues to grow worldwide, especially in low- and middle-income countries. The demand for rehabilitation services does, however, already exceed their availability, leaving needs unmet (2).

Given the restricted resources and facing an increasing demand requires the use of the available resources efficiently and further development of healthcare services in a way that promotes the greatest clinical benefit combined with the lowest risk of harm and a high degree of acceptability to those in need for healthcare.

Indeed, rehabilitation services promote autonomy and participation as, e.g., has been shown for multidisciplinary stroke units (3). Such services apply complex combinations of specific interventions that all may contribute to the overall outcome to a variable extent. Here, evidence from high-quality clinical research can guide clinical decision-making for the benefit of those being taken care of.

Indeed, quality of healthcare can be improved when the best external evidence available is integrated in clinical decision-making in a systematic explicit manner. With the rapid expansion of clinical evidence, the opportunities for evidence-based high-quality healthcare increases. Paradoxically, the likelihood of any one person to get a complete and balanced picture of the evidence available decreases.

Systematic reviews and meta-analysis that provide a synthesis of the available evidence, e.g., for a specific intervention, can provide more precise estimates of therapeutic effects and provide more refined information for clinical decision-making, e.g., by sub-group analyses (4). Aside from the expanding clinical trial evidence, there is also rapidly increasing systematic review and meta-analyses evidence available for rehabilitation interventions. A PubMed search for stroke rehabilitation meta-analyses revealed a total of 220 references for the two decades from 1993 to 2013, but 522 references for the last 8 years only (https://pubmed.ncbi.nlm.nih.gov/?term=stroke+rehabilitation&filter=pubt.meta-analysis&filter=years.2014-2021; accessed April 24, 2021). Hence, for many healthcare questions, such evidence can be used as a “primary source” for clinical decision-making.

Cochrane's systematic reviews provide high-quality information to support informed decision-making, i.e., they provide the current status of evidence in a valid, critically appraised and comprehensive way. They do, however, not provide practice recommendations based on this evidence.

The same holds true for the many other sources of systematic reviews and meta-analyses available in the literature. In addition, there is a risk that informed decision-making could even be misled by such information. When systematic reviews and meta-analyses are based on poor methodology, they are at risk to provide aggregated data that are invalid, and thus, any recommendations based on such information would be at risk to be invalid.

Yet, it would be important or even mandatory to integrate valid up-to-date therapeutic information in clinical decision-making and thereby improve global health.

Several barriers exist that might reduce the chance for an adequate integration of Cochrane and other systematic review evidence into clinical practice:

1. Clinicians, their patients, and other stakeholders might, in many instances, have difficulties to apprehend a systematic review and its direct implications for clinical practice. They might be uncertain when it comes to implications for clinical decision-making based on Cochrane and other evidence. This can be caused by many reasons, one being that Cochrane reviews and other systematic reviews are complex reports and that their structure is not easy to follow for somebody not trained to do so.

2. Not all systematic reviews that are relevant for a healthcare question fulfill the high-quality standards of Cochrane reviews. While systematic reviews can provide a valid summarizing picture of the currently available best evidence, they are at risk for flaws, invalid meta-analyses, and thereby conclusions. Therefore, it is necessary to critically appraise systematic reviews and their meta-analyses before practice recommendations can be deduced.

3. While evidence is a fundamental aspect for decision-making in evidence-based practice, it is in itself not a recommendation. Several steps have to be taken from a body of evidence to recommendations. Recommendations need to be based on different available alternative options for treatment that usually would not be addressed in a single systematic review. Systematic reviews frequently focus on a specific intervention. Furthermore, stakeholders' views and preferences need to be considered. Weighing of benefits, side effects, risks and their overall balance, together with potential resource implications and implementation issues across healthcare settings all need to be taken into account.

Even when looking at existing healthcare guidelines many show restrictions. Many guidelines are not systematically evidence-based, or if they are, they cover only a restricted scope of healthcare questions, and apply to specific healthcare situation only, mostly as prevalent in high-income countries (5).

As a consequence, the World Federation of Neurorehabilitation (WFNR) and its Special Interest Groups (SIG) developed evidence-based practice recommendations for stroke rehabilitation (6). Here, practice recommendations for the major topics encountered in stroke rehabilitation were systematically evidence-based while they were not linked to specified healthcare situations. For that purpose, the WFNR SIG Clinical Pathways provided a methodology for both clinical appraisal of the most up-to-date external evidence available and the systematic steps to be taken from the evidence to clinical practice recommendations (6).

This methodology paper extends this practice recommendation development methodology and describes a structured process for the development and report of evidence-based clinical practice recommendations that specifically use systematic reviews and meta-analyses as evidence source. It provides a comprehensive multi-step outcome-centered framework for the development and reporting of such practice recommendations based on a combination of internationally agreed methods. An added distinctive feature of the methodology is to focus on the most up-to-date most valid evidence and hence to support the development of valid practice recommendations without investing too much time for the critical appraisal of less informative and/or less valid evidence.

## Materials

### General Methodological Requirements

The general methodology is used as described by Appraisal of Guidelines for REsearch & Evaluation (AGREE) II (7, 8). The domains to be considered for the practice recommendation development process are presented in this section.

#### Abstract and Plain Language Summary

The recommendations provide both a structured abstract (purpose, methods, evidence synthesis, and practice recommendations) and a plain language summary (purpose, development characteristics, key results, and practice recommendations).

#### Scope and Purpose

The description of scope and purpose of the recommendations includes

- the overall objective,
- the health question addressed, and
- a specification of the target population, condition(s), intervention(s) or exposures, comparison(s), outcome(s), and healthcare setting(s) of interest.

A clear description of the population (i.e., patients, public, etc.) covered should be provided. The age range, sex, clinical description, and comorbidity may be provided.

#### Stakeholder Involvement

##### Practice Recommendation Developer Group

The practice recommendation development group should include individuals from all relevant professional groups. This may include members of the steering group, the research team involved in selecting and reviewing/rating the evidence, and individuals involved in formulating the final recommendations.

For each member of the guideline development group, the following information is included:

- name,
- discipline/content expertise (e.g., neurosurgeon, methodologist),
- institution (e.g., St. Peter's hospital),
- geographical location (e.g., Seattle, WA), and
- description of the member's role in the guideline development group.

##### Integration of Views and Preferences of the Target Population

A statement of type of strategy, the data acquisition or search strategy used to capture patients'/public's views and preferences (e.g., participation in the guideline development group, literature review of values and preferences), and the outcomes/information gathered on patient/public information should be given.

#### Practice Recommendation Target User Description

A clear description of intended practice recommendation audience (e.g., specialists, family physicians, patients, and clinical or institutional leaders/administrators) and a description of how the recommendation may be used by its target audience (e.g., to inform clinical decisions, to inform policy, to inform standards of care) should be documented.

### Critical Appraisal of Systematic Reviews

Since, for any health question, both high-quality Cochrane reviews and/or other systematic reviews might contribute to the evidence synthesis, it is important to critically appraise systematic reviews before their results can be used for practice recommendations. AMSTAR (A MeaSurement Tool to Assess systematic Reviews) is a valid, reliable, and useable instrument that helps users differentiate between systematic reviews, focusing on their methodological quality and expert consensus (9) (https://amstar.ca/index.php; accessed April 24, 2021).

### Methodology for a Systematic Link Between Evidence and Practice Recommendations

The Grading of Recommendations Assessment, Development, and Evaluation (GRADE) working group developed a common, sensible, and transparent approach to grading both the quality (or certainty) of evidence and the strength of recommendations (10) (http://www.gradeworkinggroup.org/ accessed April 24, 2021).

Many international organizations have provided input into the development of the GRADE approach, which is now considered the standard in guideline development.

According to GRADE, each of the four GRADE criteria for determining the strength of a recommendation (the balance of desirable and undesirable consequences, quality of evidence, values and preferences of those affected, and resource use) explicitly needs to be taken into consideration. The general approach used should be reported (e.g., if and how costs were considered, whose values and preferences were assumed, etc.). The strength of recommendation for or against a specific management option should be expressed using two categories (weak or strong recommendation) and the definitions/interpretation for each category should be consistent with those used by the GRADE Working Group.

Since the GRADE approach is used to judge the quality of evidence in Cochrane systematic reviews (4), the systematic approach for determining the strength of recommendations suggested by GRADE is methodologically in good agreement with Cochrane evidence. For other systematic reviews and meta-analyses, it can equally be applied if not an integral part of that work.

### Combining Methods for Practice Recommendation Developments

This methodology paper makes use of the Cochrane methodology to assess, synthesize, and report evidence (4), the GRADE methodology for rating quality of evidence and for determining the strength of recommendations (10), as well as the guideline evaluation methodology as described and postulated by AGREE.

All these methodological approaches that are compatible with each other are explicitly used. They are not modified for the purpose presented in this methodology paper.

Based on the strength of all three approaches, a methodology is described on how to develop evidence-based practice recommendations using Cochrane reviews and other systematic reviews.

This approach does not neglect the need to systematically base practice recommendations on reports of experimental and/or observational clinical studies (11). It is at the discretion of stakeholders to define the relevant source of information for each healthcare question they address.

It does, however, suggest a systematic approach from evidence to clinical decision in case information from systematic reviews and meta-analyses can be used. The methodology suggested is primarily described for a comprehensive use of meta-analytic data for a given healthcare question (where this is available).

Any results of this type of work can be used by different stakeholders such as healthcare professionals, patients, healthcare organizations, or guideline developers.

The presented methodology can also be used to provide guidance based on a single systematic review, i.e., to make clinical decision implications transparent for a given piece of information. Implications in terms of lack of comprehensiveness (when not based on a systematic search, but rather a single systematic review at hand, e.g., just published) should explicitly be stated in a report.

The proposed methodological approach is thus based on the original work published by these groups (as stated above) and used for a given purpose, i.e., to develop evidence-based recommendations for certain health questions by generating a systematic link from evidence to practice recommendations.

## Methods

### Systematic Search

For each systematic search, its strategy based on search terms used, sources consulted, and dates of the literature covered should be documented. Electronic databases (e.g., MEDLINE, EMBASE, and CINAHL), databases of systematic reviews (e.g., the Cochrane Library and DARE), handsearching journals, and reviewing conference proceedings can be sources.

The information provided should include the following:

- named electronic database(s) or evidence source(s) where the search was performed (e.g., MEDLINE, EMBASE, PsychINFO, and CINAHL);
- time periods searched (e.g., January 1, 2011, to April 30, 2021);
- search terms used (e.g., text words, indexing terms, and subheadings); and
- full search strategy included (e.g., possibly located in appendix).

Other guidelines (e.g., the US National Guideline Clearinghouse, the German Guidelines Clearinghouse) can serve as a valuable reference for comparison, but rarely as a primary source.

### Criteria and Methods for Evidence Selection

Criteria for including/excluding evidence, e.g., systematic reviews identified by the search, should be provided. These criteria should be explicitly described, and reasons for including and excluding evidence should be clearly stated. For example, guideline authors may decide to only include evidence from randomized clinical trials and to exclude articles not written in English.

A description of the eligibility criteria includes the target population characteristics, study design, comparisons, outcomes, language, and context.

In addition, the evidence to decision process needs to be based on the most up-to-date valid evidence. Therefore, a decision is taken to select among systematic reviews and meta-analyses that cover the same comparison for a given outcome the one high-quality review with the broadest coverage, i.e., the most recent review including the largest population of participants for the EtD process. In that case, the other reviews can be excluded from the data extraction process (“secondary exclusion” as compared to “primary exclusion” by exclusion criteria). In case the two dimensions (validity of the review and its coverage) are not congruent, case-by-case judgment is recommended to select the most informative valid information (review). It needs to be taken into account that this evidence selection procedure has a rather specific focus. A review that does not have the broadest coverage for one outcome (e.g., therapeutic effect on body function) of interest might well do so for another outcome of interest (e.g., therapeutic effect on activities) and would then be used as evidence source for that aspect. Overall, the evidence selection process is outcome-centered.

There should be at least two independent assessors for study selection. A consensus process should be used when disagreement arises in study selection.

A list of included studies and a list of excluded potentially relevant studies and justification of their exclusion should be given. Non-inclusion of evidence may be necessary for a range of reasons such as inappropriate/irrelevant populations, study design, interventions, comparisons, outcomes, or healthcare settings. “Primary” exclusion should, however, not be based on risk of bias, which is dealt with separately and later in the review process.

### Data Extraction and Management

There should be at least two independent assessors for data extraction. A consensus process should have been used when disagreements arose in data extraction.

### Methods Used to Assess the Strengths and Limitations of the Body of Evidence and Methods Applied for Best Evidence Synthesis

The methods to assess the quality of included (systematic) reviews and the quality of evidence of clinical trials included in these reviews as well as the methods used for best evidence synthesis should be described. Recommendations for such methods are given below (section Synthesizing Evidence and Developing Recommendations).

### Methods Used for Formulating the Recommendations

An explicit link between the recommendations and the evidence on which they are based should be included in the practice recommendation. The detailed methods recommended for this process are given below.

In addition, a description of the methods used to formulate the recommendations and how final decisions were arrived at should be provided. For example, methods may include a voting system, informal consensus, and formal consensus techniques [e.g., Delphi, Glaser techniques; (12)]. Areas of disagreement and methods of resolving them should be specified.

### External Review of the Recommendations

Practice recommendations should be reviewed externally before they are published. Reviewers should not have been involved in the guideline development group. It is preferable that both experts in the clinical area and some methodological experts are involved. Target population (patients and the public) representatives may also be included.

The description of the external review process includes:

- purpose and intent of the external review (e.g., to improve quality, gather feedback on draft recommendations, assess applicability and feasibility, and disseminate evidence);
- methods taken to undertake the external review (e.g., rating scale and open-ended questions);
- description of the external reviewers (e.g., number, type of reviewers, and affiliations);
- outcomes/information gathered from the external review (e.g., summary of key findings); and
- description of how the information gathered was used to inform the guideline development process and/or formation of the recommendations (e.g., guideline panel considered results of review in forming final recommendations).

### Clarity of Presentation

A recommendation should provide a concrete and precise description of which healthcare option is appropriate in which situation and population group based on the body of evidence. In case of uncertainty, this should also be stated.

Practice recommendations that target the management of a disease should consider the different possible options for screening, prevention, diagnosis, or treatment of the condition they cover. These possible options should be clearly presented with a description of options and of population or clinical situation most appropriate to each option.

Users should be able to find the most relevant recommendations easily.

### Applicability

Existing facilitators and barriers that will impact the application of recommendations along with advice and/or tools on how the recommendations can be put into practice should be provided.

The potential resource implications of applying the recommendations need to be considered, and suggestions for monitoring and/or auditing criteria (such as process measures, behavioral measures, and clinical or health outcome measures) should be provided.

### Editorial Independence

The formulation of recommendations should not unduly be biased with competing interests.

Therefore,

- the name of the funding body or source of funding (or explicit statement of no funding) and a statement that the funding body did not influence the content of the report should be given; and
- competing interests of development group members need to be recorded and addressed including
  - a description of the types of competing interests considered,
  - methods by which potential competing interests were sought,
  - a description of the competing interests, and
  - a description of how potentially competing interests were managed.

### Procedure for Updating the Practice Recommendations

Practice recommendations need to reflect the current research. A clear statement about the procedure for updating the recommendations should be provided.

### Synthesizing Evidence and Developing Recommendations

This part of the methods section has primarily been based on the methodology as described in the GRADE Handbook (10) and the methodological assessment of systematic reviews as described in AMSTAR (9).

#### The Healthcare Question

Recommendations should answer focused healthcare questions. A sensible way to frame a healthcare question is the PICO framework (patient, intervention, comparison, outcome). Any evidence needs to be reflected against the PICO framework set forth for the practice recommendations to be developed (healthcare question addressed) and the respective PICO framework of a systematic review.

Two different formats for questions about management can be used:

- Should [intervention] vs. [comparison] be used for [health problem]?
- Should [intervention] vs. [comparison] be used in [population]?

As well as one format for questions about diagnosis:

- Should [intervention] vs. [comparison] be used to diagnose [target condition] in [health problem and/or population]?

With regard to patient populations, it has to be taken into account whether populations are sufficiently homogeneous that interventions apply to them in a comparable way. Otherwise, sub-populations should be specified.

For practice recommendations, it is important to consider all outcomes that are important or critical for decision-making, especially including all patient-important outcomes; outcomes of limited importance do not have to be included (mostly are not). The determination of relevant outcomes should be related to patients' values and preferences regarding the intervention(s) in question.

In many instances, a healthcare intervention is designed to improve a health condition. Any recommendation should be based on the fact that there is evidence to suggest that the intervention does change the health condition of interest, to what degree it does so (magnitude of effect), and with which certainty. For such an instance, a precondition for a recommendation would be that there is (biologically plausible) evidence that therapy A improves health condition B (e.g., a body function). Patient-important outcomes can, at times, measure different constructs, e.g., whether disability, health-related quality of life, and participation in social activities are improved by a healthcare intervention for the condition they suffer from. The recommendation should put considerable weight on these findings. Both the causal relationship between an intervention and the improvement of a health condition, and the therapeutic impact on patient-important outcomes are relevant for clinical decision-making.

Furthermore, both benefit and harm need to be assessed. A harmful incident (adverse event) is an incident that results in harm to a patient (e.g., the wrong unit of blood was infused and the patient died from a haemolytic reaction). Harm is constituted by disease, injury, and suffering that may be physical, social, or psychological disability, or death (13). Disease is a physiological or psychological dysfunction. Injury is damage to tissues caused by an agent or event, and suffering is the experience of anything subjectively unpleasant. Suffering includes pain, malaise, nausea, depression, agitation, alarm, fear, and grief. Disability implies any type of impairment of body structure or function, activity limitation, and/or restriction of participation in society, associated with past or present harm.

The degree of harm is as follows:

- None—patient outcome is not symptomatic or no symptoms detected and no treatment is required.
- Mild—patient outcome is symptomatic, symptoms are mild, loss of function or harm is minimal or intermediate but short term, and no or minimal intervention (e.g., extra observation, investigation, review, or minor treatment) is required.
- Moderate—patient outcome is symptomatic, requiring intervention (e.g., additional operative procedure; additional therapeutic treatment), an increased length of stay, or causing permanent or long-term harm or loss of function.
- Severe—patient outcome is symptomatic, requiring life-saving intervention or major surgical/medical intervention, shortening life expectancy, or causing major permanent or long-term harm or loss of function
- Death—on balance of probabilities, death was caused or brought forward in the short term by the incident.

It is suggested that all these aspects are specifically addressed and the overall practice recommendation is based on the combined evaluation of the evidence.

Furthermore, third-party relevant outcomes might be important as well.

Similarly, all relevant therapeutic alternatives need to be entertained (including those that might apply to different relevant healthcare settings).

#### Quality of Included Systematic Reviews

Systematic reviews are subject to a range of biases and increasingly include non-randomized studies of intervention (NRSI). It is important to critically appraise the quality of systematic reviews when their evidence is supposed to be used in the development of practice recommendations. AMSTAR 2 is a critical appraisal tool for systematic reviews that include randomized or non-randomized studies of healthcare interventions, or both (9). It is used in this context to assess the quality of included systematic reviews in a standardized systematic way.

AMSTAR 2 rates 16 different quality aspects [cited in abbreviated form Supplementary Appendix 1: AMSTAR 2 guidance document; (9)]:

Item 1: Did the research questions and inclusion criteria for the review include the components of PICO? To score “Yes,” appraisers should be confident that the four elements of PICO are described somewhere in the report.

Item 2: Did the report of the review contain an explicit statement that the review methods were established prior to conduct of the review and did the report justify any significant deviations from the protocol?

To score “Yes,” authors should demonstrate that they worked with a written protocol with independent verification (by a registry or another independent body, e.g., research ethics board or research office) before the review was undertaken.

Item 3: Did the review authors explain their selection of the study designs for inclusion in the review?

The selection of study types for inclusion in systematic reviews should not be arbitrary. To justify restriction of the review to randomized controlled trials (RCTs), the authors should argue that they can provide a complete picture of the effects they are interested in. Restriction of a review to only NRSI is justified when RCTs cannot provide the necessary outcome data, or in the case where reviews of RCTs have been completed and the review of NRSI will complement what is already known. Inclusion of both RCTs and NRSI may be justified to get a complete picture of the effectiveness and harms associated with an intervention.

Item 4: Did the review authors use a comprehensive literature search strategy?

At least two bibliographic databases should be searched. The report should include years and databases examined (e.g., Central, EMBASE, and MEDLINE). Keywords and/or MESH terms should be reported and the full search strategy should be available on request. Publications in all relevant languages should be sought and a justification should be provided when there are language restrictions. Where the gray literature is considered important, authors should have searched appropriate sources, such as trial registries, conference abstracts, dissertations, and unpublished reports on personal websites (e.g., universities and ResearchGate). In addition, trials of medical interventions may not have been published in peer-reviewed journals but can be obtained directly from company sponsors or directly from investigators. To score “Yes,” appraisers should be satisfied that all relevant aspects of the search have been addressed by review authors.

Item 5: Did the review authors perform study selection in duplicate?

Best practice requires two review authors to determine eligibility of studies for inclusion in systematic reviews. A consensus process should have been used when disagreements arose in study selection. If one individual carried out selection of all studies, with a second reviewer checking agreement on a sample of studies, it is recommend that a kappa score indicating “strong” agreement (0.80 or greater) should have been achieved.

Item 6: Did the review authors perform data extraction in duplicate?

As in Item 5, there should have been at least two independent assessors performing data extraction. A consensus process should have been used when disagreements arose. In the event that one individual carried out data extraction, a second reviewer should have checked agreement on a sample of studies, and they should have achieved a kappa score of 0.80 or greater.

Item 7: Did the review authors provide a list of excluded studies and justify the exclusions?

This item requires review authors to provide a complete list of potentially relevant studies with justification for the exclusion of each one.

Item 8: Did the review authors describe the included studies in adequate detail?

The description of subjects, interventions, controls, outcomes, design, analysis, and settings of the studies should be provided. The detail should be sufficient for an appraiser, or user, to make judgments about the extent to which the studies were appropriately chosen (in relation to the PICO structure) and whether the study populations and interventions were relevant to their own practice or policy.

Item 9: Did the review authors use a satisfactory technique for assessing the risk of bias (RoB) in individual studies that were included in the review?

This is a crucial part of the appraisal of any systematic review, particularly those that include NRSI. The key appraisal question is whether review authors have taken account of the risk of bias when summarizing and interpreting the results.

Whatever instrument was used by the review authors, appraisers should be satisfied that it addresses the items listed in item 9 of the instrument.

Item 10: Did the review authors report on the sources of funding for the studies included in the review?

Several investigations have shown that commercially sponsored studies are more likely to have findings that favor a sponsor's product than independently funded studies. It is valuable for review authors to document the funding sources for each study included in the review or to record that the information was not provided in the study reports.

Item 11: If meta-analysis was justified, did the review authors use appropriate methods for statistical combination of results? (Only complete this item if meta-analysis of other data synthesis techniques were reported.)

Review authors should have stated explicitly in the review protocol the principles on which they based their decision to perform meta-analysis of data from the included studies. These include the desire to obtain a single pooled effect (for instance, from a number of compatible but underpowered studies) and the extent to which the studies are compatible (in terms of populations controls and interventions) and therefore capable of being combined.

Where meta-analysis was considered appropriate, authors should have explained their decisions to use fixed or random effects models in the case of RCTs, and set out the methods they intended to use to investigate heterogeneity.

If results from large NRSIs are combined with those from smaller RCTs, the pooled estimates of effect will be dominated by the data from the nonrandomized studies. In addition, the results from NRSI may be affected by a range of biases (see above), meaning that the overall pooled estimates may be precise but biased. It is rare for a NRSI to have as low risk of bias as a high-quality RCT of the same research question and confidence intervals for NRSI (and pooled estimates based on NRSI) should be viewed with caution. Review authors should therefore report pooled estimates separately for the different study types.

Furthermore, when combining the results of NRSI, review authors should pool the fully adjusted estimates of effect, not the raw data. If they do the latter, there should be a clear justification.

Item 12: If meta-analysis was performed, did the review authors assess the potential impact of RoB in individual studies on the results of the meta-analysis or other evidence synthesis?

In cases where review authors have chosen to include only high-quality RCTs there may be little discussion of the potential impact of bias on the results, but where they have included RCTs of variable quality, they should assess the impact of this by regression analysis, or by estimating pooled effect sizes with only studies at low ROB. In the case of NRSI, they should estimate pooled effect sizes while including only studies at low or moderate risk of bias, and/or only those at low ROB (if there are any). If meta-analyses (or other data synthesis techniques such as regression analysis) were not performed, the authors should still provide some commentary on the likely impact of ROB on individual study results.

Item 13: Did the review authors account for RoB in individual studies when interpreting/discussing the results of the review?

Even if meta-analyses were not conducted, review authors should include discussion of the impact of ROB in the interpretation of the results of the review. This is always important, but especially when reviews include RCTs with variable ROB, and with any review that includes NRSI. This discussion should not be limited to the impact of ROB on the pooled estimates (see above), but should also consider whether it may account for differences between the results of individual studies.

Item 14: Did the review authors provide a satisfactory explanation for, and discussion of, any heterogeneity observed in the results of the review?

There are many more potential causes of heterogeneity in the results of NRSI than in RCTs. Many factors were considered in this instrument, including different study designs, different methods of analysis, different populations, and differing intensities of the intervention(s)—dosages in the case of drugs. Both the PICO elements and the domains of bias listed in Item 9 should also be considered as important potential sources of heterogeneity in the results. Review authors should explore these possibilities and discuss the impact of heterogeneity on the results conclusions and any recommendations.

Item 15: If they performed quantitative synthesis, did the review authors carry out an adequate investigation of publication bias (PB) (small study bias) and discuss its likely impact on the results of the review?

This is a very important issue, but can be difficult for review authors and appraisers to resolve completely. Typically, statistical tests or graphical displays are used, and if they are positive, then it indicates the presence of PB. However, negative tests are not a guarantee of the absence of PB as the tests are insensitive. To some extent, the importance of PB depends on context and setting.

Item 16: Did the review authors report any potential sources of conflict of interest, including any funding they received for conducting the review?

As noted above (under ROB), individual studies funded by vested interests may generate results that are more likely to favor the intervention than do independent studies. The same assumption applies to systematic reviews, and authors should report their direct funding sources. Journals generally will require this. However, assessment of the reviewers' conflicts of interest does not stop there. They should report their other ties. The review may be independently funded, but the authors have ties to companies that manufacture products included in the systematic review. Professional conflicts of interest are powerful, but harder to discern as they are seldom reported. When investigators have a career-long investment in a field of research, a review that conflicts with their long-held beliefs can be confronting. Potential conflicts of interest of this type will be hard to assess but may be inferred from the fact that the reviewers have published extensively in the field being reviewed and their studies are included in the systematic review. While it can be argued that the effects of competing interests might manifest as flaws in the other domains, this item should nevertheless always be rated separately.

When *I*^2^ statistics are used, a rough guide for the interpretation and verbal description of the level of heterogeneity can be (14):

- 0–29%: low heterogeneity, might not be important;
- 30–49%: may represent moderate heterogeneity^*^;
- 50–74%: may represent substantial heterogeneity^*^; and
- 75–100%: considerable heterogeneity^*^.

^*^The importance of the observed value of *I*^2^ depends on (1) magnitude and direction of effects, and (2) strength of evidence for heterogeneity (e.g., *P*-value from the χ^2^- test, or a confidence interval for *I*^2^: uncertainty in the value of *I*^2^ is substantial when the number of studies is small).

#### Quality of Evidence in Included Reviews According to GRADE

The quality of evidence reflects the extent to which our confidence in an estimate of the effect is adequate to support a particular recommendation. The quality of evidence is rated for each outcome across studies (i.e., for a body of evidence). This does not mean rating each study as a single unit. Rather, GRADE is “outcome centric”; rating is done for each outcome, and quality may differ—indeed, is likely to differ—from one outcome to another within a single study and across a body of evidence.

In case a review included in the “evidence to decision” process presents a GRADE quality of evidence rating this will be considered, yet critically appraised, and in case the information available indicates a more valid rating in the specific context modified accordingly. Such deviations should be noted.

### GRADE Definition for Quality of Evidence

High—We are very confident that the true effect lies close to that of the estimate of the effect.

Moderate—We are moderately confident in the effect estimate: The true effect is likely to be close to the estimate of the effect, but there is a possibility that it is substantially different Low—Our confidence in the effect estimate is limited: The true effect may be substantially different from the estimate of the effect.

Very low—We have very little confidence in the effect estimate: The true effect is likely to be substantially different from the estimate of effect.

Quality of evidence is a continuum; any discrete categorization involves some degree of arbitrariness. Nevertheless, advantages of simplicity, transparency, and vividness outweigh these limitations.

In the GRADE approach to quality of evidence:

- randomized trials without important limitations provide high-quality evidence, and
- observational studies without special strengths or important limitations provide low-quality evidence.

Limitations or special strengths can, however, modify the quality of the evidence of both randomized trials and observational studies.

**Table:** 

*Factors reducing the quality of the evidence*	*Factor Consequence*
Limitations in study design or execution (risk of bias)	↓ 1 or 2 levels
Inconsistency of results	↓ 1 or 2 levels
Indirectness of evidence	↓ 1 or 2 levels
Imprecision	↓ 1 or 2 levels
Publication bias	↓ 1 or 2 levels
*Factors increasing the quality of the evidence*	*Factor Consequence*
Large magnitude of effect	↑ 1 or 2 levels
All plausible confounding would reduce the demonstrated effect	
or increase the effect if no effect	
was observed	↑ 1 level
Dose–response gradient	↑ 1 level

Limitations in study design or execution (risk of bias) in RCTs may include inadequate randomization, lack of allocation concealment, lack of blinding leading to performance and/or detection bias, attrition bias, selective reporting bias, and other types of bias.

For a more refined explanation of risk of bias and other factors modifying the quality of the evidence, see the GRADE Handbook (10) and the Cochrane Handbook (4).

All abovementioned factors need judgment; they can be combined, yet they are not strictly additive. Up- or downgrading rests on the assessment that limitations are serious and there is a substantial risk of bias for the results. Reasons for (and at times for not) down- or upgrading should be provided (e.g., in footnotes).

Recommendations are based on evidence for interventions and certain outcomes across studies and potentially across systematic reviews. Thus, there is a need to critically appraise the quality of evidence across clinical trials and at times across systematic reviews. In deciding the overall quality of evidence (across studies and reviews), the contribution of individual studies, e.g., based on sample size and number of outcome events needs to be taken into consideration.

### The GRADE Evidence Profile

Two independent assessors should extract the information and perform all related judgments. A consensus process should be used when disagreements arise in information extraction and judgments are declared in advance.

A GRADE evidence profile is particularly useful for the presentation of evidence supporting a clinical practice recommendation.

It is augmented by a commentary of the validity assessment of the systematic review used for the outcome-related evidence (based on AMSTAR 2).

The evidence profile is the first part of the EtD table that in addition to the evidence part includes further aspects that are taken into consideration before an overall recommendation for a type of therapy is given. The latter reasoning will be presented below (paragraph “GRADE recommendations”).

The standard format for the evidence profile includes:

- A list of primary and secondary outcomes, and for each outcome and analysis:
- the reference (e.g., Miller et al., 2018);
- the study design (including PICO information);
- number of studies and participants;
- relevant limitations of the review as indicated by the critical appraisal tool for systematic reviews (AMSTAR 2);
- rating (GRADE) of the quality of evidence for each outcome (which may vary by outcome) and reasons why the rating was up- or downgraded, i.e., risk of bias (with specific qualifiers such as random sequence generation, allocation concealment etc.), imprecision, heterogeneity, indirectness, imprecision, publication bias, magnitude of effect, dose–response relationship, and other considerations;
- the assumed risk; a measure of the typical burden of the outcomes, i.e., illustrative risk or also called baseline risk, baseline score, or control group risk;
- the corresponding risk; a measure of the burden of the outcomes after the intervention is applied, i.e., the risk of an outcome in treated/exposed people based on the relative magnitude of an effect and assumed (baseline) risk;
- the relative effect; for dichotomous outcomes, the table will usually provide risk ratio, odds ratio, or hazard ratio;
- the absolute effect; for dichotomous outcomes, the number of fewer or more events in treated/exposed group as compared to the control group; for ratio scaled outcomes, standardized mean differences or mean differences;
- a verbal description of the therapeutic effect and uncertainties regarding its estimate;
- indication whether the evidence for an outcome favors or discourages the decision for a therapy; and
- footnotes, if needed, to provide explanations about information in the table such as elaboration on judgments about the quality of evidence.

Symbols used to indicate whether the evidence for a given outcome favors or discourages the decision for a therapy are:

++ clearly favors therapy
+ favors therapy somewhat
0 does not favor or discourage use of therapy
- discourages use of therapy
– clearly discourages use of therapy

#### GRADE Recommendations

A recommendation reflects the extent to which the group developing the recommendation is confident that desirable effects of an intervention outweigh undesirable effects in case of a positive recommendation, or that undesirable effects of an intervention outweigh desirable effects in case of a negative recommendation.

The recommendation implies that it is valid across the range of patients for whom the recommendation is intended.

Even though the balance between desirable and undesirable effects of an intervention reflects a continuum, GRADE specifies two categories of strength of recommendation, i.e., a weak or a strong recommendation in favor or against an intervention.

For a strong recommendation, it is necessary to be certain about the various factors that influence the strength of recommendation and to have the information at hand that support a clear balance toward either the desirable or the undesirable effects of an intervention. When the information is such that the desirable effects of an intervention probably outweigh the undesirable effects (or vice versa), but appreciable uncertainty exists, a weak recommendation for (or against) an intervention is warranted.

Various domains contribute to the strength of a recommendation:

- Balance between desirable and undesirable outcomes: The larger the difference between the desirable and undesirable outcomes and the more important an outcome is based on estimated typical values and preferences, the more likely a strong recommendation is warranted. For the judgment of magnitude of effect, both measures of effect size (comparable across outcome measures) and judgments of the absolute effects, e.g., compared to the minimally important difference (MID) for an outcome measure, are suggested.
- Quality of evidence: The higher the quality of evidence and hence the confidence in the magnitude of effect, the more likely a strong recommendation is warranted.
- Values and preferences of those affected: The greater the confidence that the observed effects (invariably) apply to the values and preferences of patients, the more likely a strong recommendation is warranted.
- Resource use: The less resources are used for the implementation of an intervention, the more likely a strong recommendation is warranted.

With regard to resource use, a decision whether or not to consider resource use, and if so, its integration in the recommendation, the perspective taken (e.g., from an individual out of the pocket perspective to a societal perspective including all important resource implications), any differences in resource use between intervention and control, and the evidence for incremental cost should be taken into consideration and made transparent.

While this guidance frequently applies, there are instances where recommendations are and need to be taken on different grounds. GRADE has identified several situations when a strong recommendation is warranted in spite of low or very low quality of evidence:

1. When low-quality evidence suggests benefit in a life-threatening situation (evidence regarding harms can be low or high).
2. When low-quality evidence suggests benefit and high-quality evidence suggests harm or a very high cost.
3. When low-quality evidence suggests equivalence of two alternatives, but high-quality evidence of less harm for one of the competing alternatives.
4. When high-quality evidence suggests equivalence of two alternatives and low-quality evidence suggests harm in one alternative.
5. When high-quality evidence suggests modest benefits and low/very-low-quality evidence suggests possibility of catastrophic harm.

It is an option to recommend using interventions only in research, if

- the evidence thus far is insufficient to support a decision for or against an intervention,
- further research has a high potential to reduce this uncertainty, and
- and if it is thought to be of good value for the anticipated costs.

The final recommendation made by the development group is a consensus based on the judgments of the group members, informed by the evidence presented and the group members' expertise and experience.

- Recommendations ideally are accompanied by
- plain language phrasing of the recommendation,
- the justification for the recommendation (report on the decisions taken about the strength of recommendation),
- specific implementation aspects related to subgroups,
- implementation issues related to acceptability and feasibility,
- suggestions for monitoring and evaluation of its implementation, and
- any uncertainties that warrant further research.

Along these lines, generic EtD tables can be used and facilitate decision-making, record judgments, and document the process of going from evidence to the decision.

They typically describe

- whether there is a priority problem,
- the benefits and harms of the options,
- the certainty how much people value the main outcome,
- the size of desirable and undesirable effects and their balance,
- associated resource use,
- the incremental resource use relative to the net benefit,
- the impact on health inequities,
- the acceptability of options,
- the feasibility,
- the recommendation,
- its justification,
- subgroup considerations,
- implementation considerations,
- monitoring and evaluation considerations, and
- implications for research priorities.

Symbols used to indicate the strength of recommendation:

↑↑: strong recommendation for an intervention
↑: weak recommendation for an intervention
↓: weak recommendation against an intervention
↓↓: strong recommendation against an intervention

The strength of recommendations has different implications for patients, healthcare professionals, and other stakeholders as illustrated next.

**Table:** 

	Strong recommendation	Weak recommendation
Patient	Most individuals would want the intervention	The majority would want the intervention, but many would not
Healthcare professional	Most individuals will receive the intervention; adherence to the recommendation might be used as quality indicator	Different choices are appropriate for different patients; more time allocation for informed decision taking expected
Policymaker	Recommendation can be used for policymaking and performance indicators	Policymaking requires debate and involvement of stakeholders and is likely to vary according to circumstances

It is important to note, however, that recommendations never serve as dictates. Even strong recommendations based on high-quality evidence will not apply to all circumstances and all patients.

## Results

Results of the EtD process for practice recommendation projects are:

1. A protocol before the work commences, and
2. A report on evidence-based practice recommendations once the work is done.

### Framework for a Protocol

Prior to the development of evidence-based practice recommendations, a protocol should be developed, agreed on by the working group (and significant others as indicated), and made publicly available before the work commences. The suggested framework is given below.

1. Scope and purpose
  a. Objective
  b. Health question
  c. Population, condition(s), intervention(s), outcome(s), and healthcare setting(s) of interest
2. Stakeholder Involvement
  a. Practice recommendation developer group
  b. Integration of views and preferences of the target population
  c. Practice recommendation target user description
3. Methods for evidence synthesis and recommendation development
  a. Systematic search
  b. Criteria and methods for evidence selection
  c. Data extraction and management
  d. Methods used to assess the strengths and limitations of the body of evidence
    i. quality of included reviews
    ii. quality of evidence in included reviews
  e. Methods applied for best evidence synthesis
  f. Methods used for formulating the recommendations
  g. External review of the recommendations
  h. Procedure for updating the practice recommendations
4. Editorial independence
  a. Funding
  b. Competing interests of the practice recommendation developer group

### Framework for a Report

The practice recommendation report gives all the information necessary to make the development process, the evidence, its critical appraisal, the main results, further considerations taken into account, the recommendations, and their implications transparent. The suggested framework is given below.

1. Abstract (purpose, methods, evidence synthesis, practice recommendations)
2. Plain language summary (purpose, development characteristics, key results, practice recommendations)
3. Scope and purpose
  a. Objective
  b. Health question
  c. Population, condition(s), intervention(s), outcome(s), and healthcare setting(s) of interest
4. Stakeholder Involvement
  a. Practice recommendation developer group
  b. Integration of views and preferences of the target population
  c. Practice recommendation target user description
5. Methods for evidence synthesis and recommendation development
  a. Systematic search
  b. Criteria and methods for evidence selection
  c. Data extraction and management
  d. Methods used to assess the strengths and limitations of the body of evidence
    quality of included reviews
    quality of evidence in included reviews
  e. Methods applied for best evidence synthesis
  f. Methods used for formulating the recommendations
  g. External review of the recommendations
  h. Procedure for updating the practice recommendations
6. Evidence synthesis
  a. Results of the search
  b. Description of included reviews
  c. Methodological quality of included reviews
  d. Quality of evidence in included reviews
  e. Best evidence synthesis
    —stratified by condition(s), intervention(s), outcome(s), and healthcare setting(s) of interest
7. Recommendations
  a. for clinical practice, stratified by condition(s), intervention(s), outcome(s), and healthcare setting(s) of interest
  b. for research
8. Results of external review
9. References
  a. References to included reviews
  b. References to excluded reviews (primary and secondary)
  c. Additional references
10. Editorial independence
  a. Funding
  b. Competing interests of the practice recommendation developer group
11. Appendix
  a. Search algorithm(s)
  b. Further tables
    Characteristics of included reviews
    Characteristics of excluded reviews.

## Discussion

As the number of people living with disabilities are on the rise globally (1), there is an increasing demand for rehabilitation interventions to reduce their impairments, activity limitations, and participation restrictions (2).

Evidence-based clinical practice is defined as the conscientious, explicit, and judicious use of the best evidence in making decisions about the care of individual patients (15), combined with patient's values and preferences through shared decision-making (16). By systematically integrating the best available external evidence in clinical decision-making healthcare benefits, avoiding risk of harm, and selecting acceptable interventions can all be facilitated and hence the best possible use of healthcare resources.

As not only the available evidence of clinical trials for rehabilitation interventions is rapidly increasing, but also the number of systematic reviews and meta-analyses, the potential to support healthcare decisions by evidence is greatly increased nowadays.

Yet, individual healthcare workers are facing a situation where they can no longer manage to use the best external evidence if they were not systematically supported by the development and provision of evidence-based clinical practice recommendations. Necessary steps for such developments are a systematic search for and critical appraisal of the available evidence, followed by a systematic process to deduce practice recommendations using that body of evidence (5).

A methodology for such evidence-based clinical practice recommendations development had been provided (11) and was used by a multi-professional author group to provide stroke rehabilitation practice recommendations (6).

The methods described in this paper add to this methodology for situations where systematic reviews and meta-analyses are sufficiently available to be considered a valid up-to-date representation of the best external evidence for a healthcare question.

Given the increasing number of available systematic reviews for many healthcare questions, this is and will more and more frequently be the case. In addition, different systematic reviews might contribute to our knowledge for different outcomes of interest (e.g., benefit regarding body functions, activities, or participation; harm; acceptability) that are all relevant for the generation of well-balanced practice recommendations.

For such situations, an explicit multi-step approach is suggested that includes the formulation of the healthcare question addressed, the systematic search for the evidence, its critical appraisal, the extraction and the outcome-centered presentation of the evidence, the rating of its quality, strengths and weaknesses, any further considerations relevant for decision-making, and an explicit recommendation statement along with its justification, implementation, and resource aspects, i.e., a comprehensive EtD methodology.

The methodology suggested is a combination of valid and internationally accepted methods (17) as developed by the Cochrane organization (4), GRADE (10), and AGREE (7).

If practice recommendations for major topics in clinical rehabilitation could be developed in such a systematic methodologically valid way and without restricting their applicability to specific (regional) healthcare situations, a relevant impact could be achieved for many societies.

The healthcare implications of the best available external evidence could be made transparent in a systematic, valid, and well-balanced way and become accessible for many. Healthcare workers around the globe could greatly benefit for their evidence-based clinical practice by such guidance and hence those in need for effective rehabilitation interventions. Disease-related disability could more effectively be reduced and participation could be promoted.

In addition, those in charge to set up, manage, and develop healthcare structures for rehabilitation could be guided as to which interventions should be made available in their services to support certain clinically relevant outcomes.

Hence, the resources invested to develop such guidelines could promote societal benefits both at an individual and at a structural level.

While the practice recommendations themselves would have an “international” validity, their appropriate contextualization to healthcare system realities could be done regionally. In that way, the suggested methodology is also efficient; practice recommendations would not have to be developed for each region separately.

## Author Contributions

TP designed and wrote the manuscript.

## Conflict of Interest

The author declares that the research was conducted in the absence of any commercial or financial relationships that could be construed as a potential conflict of interest.
