# Supplementary material for: Methods for the Development of Healthcare Practice Recommendations Using Systematic Reviews and Meta-Analyses
Source: Front Neurol. 2021 Jul 8;12:699968. doi: 10.3389/fneur.2021.699968 (PMC8297739; doi:10.3389/fneur.2021.699968)
Supplement: Supplementary file 1 [file Table_1.pdf]

Table . Evidence-to-Decision (EtC) table for intervention XYZ in people with XYZ

Domains of interest for recommend-dations	Review reference	Participants (inclusion /exclusion criteria)	Interven-tions (type and dosage)	Compari-son (type and dosage)	Outcome measures	Number of studies (RCTs) (t) and partici-pants (n)	Limitations of the review (including AMSTAR-2)	Quality of evidence (GRADE)	Reasons to up- or down-grade (aspects and number of levels)	Effect in control group (immediate and long-term [duration])	Effect and differential effect in experimental group (immediate and long-term [duration])	Evidence of effect	Evidence favours or discourages decision for therapy
Disease condition / impairment													
						t= n=							
						t= n=							
activities													
						t= n=							
						t= n=							
participation / quality of life													
						t= n=							
						t= n=							
harm / acceptability													
						t= n=							
						t= n=							
benefit-harm-assessment<br>subgroup considera-tions							value and acceptability<br><br>resource use and feasibility						

Recommendation for clinical practice including its strength (GRADE) and its justification			
Implementation, monitoring, and evaluation		Implication for research priorities	

Abbreviations:
n.r.: not reported   t: number of trials included, n: number of participants
With regard review methodology: POP: study population, SELECT: study selection, EXTRACT: data extraction, RoB-ASS: risk of bias assessment
For reasons to up- or downgrade quality of evidence rating use: RoB: risk of bias, R: random sequence generation, A: allocation concealment, BP: blinding of participants and personnel, BO: blinding of outcome assessment, IO: incomplete outcome, S: selective reporting, P: publication bias, Im: imprecision, H: heterogeneity In: indirectness of evidence, M: large magnitude of effect, C: effects of confounding do not question effect or no effect interpretation, D: dose-response gradient
AMSTAR 2: A MeaSurement Tool to Assess systematic Reviews   GRADE: Grading of Recommendations, Assessment, Development, Evaluation
d: GRADE rating of quality of evidence deviates from rating in source, rating in source given in ( )
MD: mean difference   SMD: standardized mean difference   CI: confidence interval RCT: randomized controlled trial

Relevance of evidence for a given outcome:

++ clearly favours therapy

+ favours therapy somewhat

0 does not favour or discourage use of therapy

- discourages use of therapy

-- clearly discourages use of therapy

Overall strength of recommendation (GRADE):

↑↑ : strong recommendation for an intervention

↑ : weak recommendation for an intervention

↓ : weak recommendation against an intervention

↓↓ : strong recommendation against an intervention
